# Extreme two-phase change of ionospheric electron temperature overshoot during geomagnetic storms

**DOI:** 10.1038/s41598-025-89602-z

**Published:** 2025-02-11

**Authors:** Artem Smirnov, Yuri Shprits, Hermann Lühr, Alessio Pignalberi, Elena Kronberg, Fabricio Prol, Chao Xiong

**Affiliations:** 1 Department of Earth and Environmental Sciences, Ludwig Maximilian University of Munich (LMU), Munich, Germany; 2 GFZ Helmholtz Centre for Geosciences, Potsdam, Germany; 3 Department of Earth, Planetary and Space Sciences, University of California Los Angeles (UCLA), Los Angeles, CA USA; 4 Instituto Nazionale di Geofisica e Vulcanologia (INGV), Rome, Italy; 5 Finnish Geospatial Research Institute (FGI), National Land Survey of Finland (NLS), Espoo, Finland; 6 School of Technology and Innovation, University of Vaasa, Vaasa, Finland; 7 Department of Space Physics, College of Electronic Information, Wuhan University, Wuhan, China

**Keywords:** Ionosphere, Electron temperature, Neural networks, Space physics, Atmospheric science

## Abstract

An intense surge in the equatorial electron temperature (T_e_) at sunrise, known as the morning T_e_ overshoot, has been one of the defining ionospheric features since its discovery early in the Space Age. Despite decades of study, the behavior of the morning overshoot during geomagnetic storms remains poorly understood. We report a two-stage response of the morning T_e_ overshoot to geomagnetic activity, uncovered by a neural network model. Electron temperatures show an initial enhancement during the storm’s main phase, followed by a drastic depletion exceeding 1000 K and disappearance of the overshoot in the recovery phase. This two-phase change aligns with the early influence of westward prompt penetration electric field, overtaken by the development of the eastward disturbance dynamo later in the storm. These electric field changes affect vertical plasma drifts that redistribute electron densities, modifying ionospheric cooling rates. Our findings provide new insights into the dynamics of one of the most widely studied ionospheric features and showcase the potential of new-generation digital twin models of near-Earth space environment to reveal previously unrecognized physical patterns.

## Introduction

Electron temperature ($T_e$) is a fundamental parameter characterizing the Earth’s ionosphere and its coupling with the neutral atmosphere, magnetosphere and solar wind^1–3^. One of the most notable features of the global $T_e$ distribution, discovered early in the Space Age, is the morning electron temperature overshoot^4,5^, which represents a rapid $T_e$ increase around the geomagnetic equator at sunrise, often in excess of 3000 K^6^. It occurs due to energy exchange between newly ionized photoelectrons and ambient thermal electrons. In regions of low plasma density, each ambient electron receives a greater share of energy compared to regions of dense plasma, making this process particularly efficient. At the equator, a unique combination of low electron density due to the downward **E × B** drift overnight and inefficient heat removal by conduction allows the morning $T_e$ overshoot to develop around 05 h of local time (LT)^6,7^. The morning overshoot typically persists until electron density increases around $\sim$09 LT and presents a global $T_e$ maximum during geomagnetically quiet times.

Climatological aspects of the morning $T_e$ overshoot, including its dependence on altitude, seasons and solar activity, have been analyzed extensively and are generally well understood^5,6,8–11^. However, few studies have investigated the dynamics of the morning overshoot during geomagnetic storms. An early theoretical study by Wang et al.^12^ proposed a negative correlation of electron temperatures in the overshoot region with the geomagnetic Kp index. Using numerical modeling, they reported the strongest $T_e$ depletions at mid-latitudes and a drop of $T_e$ by a few hundred degrees around the equator. A more recent observational study by Yang et al.^13^ reported multi-day oscillations in the morning overshoot and also suggested a negative correlation with the Kp index, although their study used daily-averaged electron temperature values and did not investigate shorter-scale variations within geomagnetic storms.

In contrast to previous studies, we report a more complex, two-phase response of the morning $T_e$ overshoot to geomagnetic storms. We develop the first global neural network (NN) model of ionospheric electron temperatures that includes geomagnetic activity dependence, based on observations by the CHAllenging Minisatellite Payload (CHAMP) mission^14^. Using the developed NN model, we identify an initial intensification of the morning overshoot during the main phase of the storm, followed by a drastic electron temperature drop and near-total disappearance of the overshoot several hours after the activity peak. Our findings suggest that the storm-time dynamics of the morning $T_e$ overshoot are determined by an initial influence of the prompt penetration electric field (PPEF) during the main phase, followed by the development of disturbance dynamo electric field (DDEF) later in the storm. These electric field changes redistribute electron density, altering cooling rates in the F-region of the ionosphere. Our results revise the previous hypothesis of a simple negative correlation with geomagnetic activity, and showcase the potential of using new-generation ionospheric models to reveal previously unrecognized physical patterns.

## Results

### Developing a digital twin model of electron temperatures

**Fig. 1:**
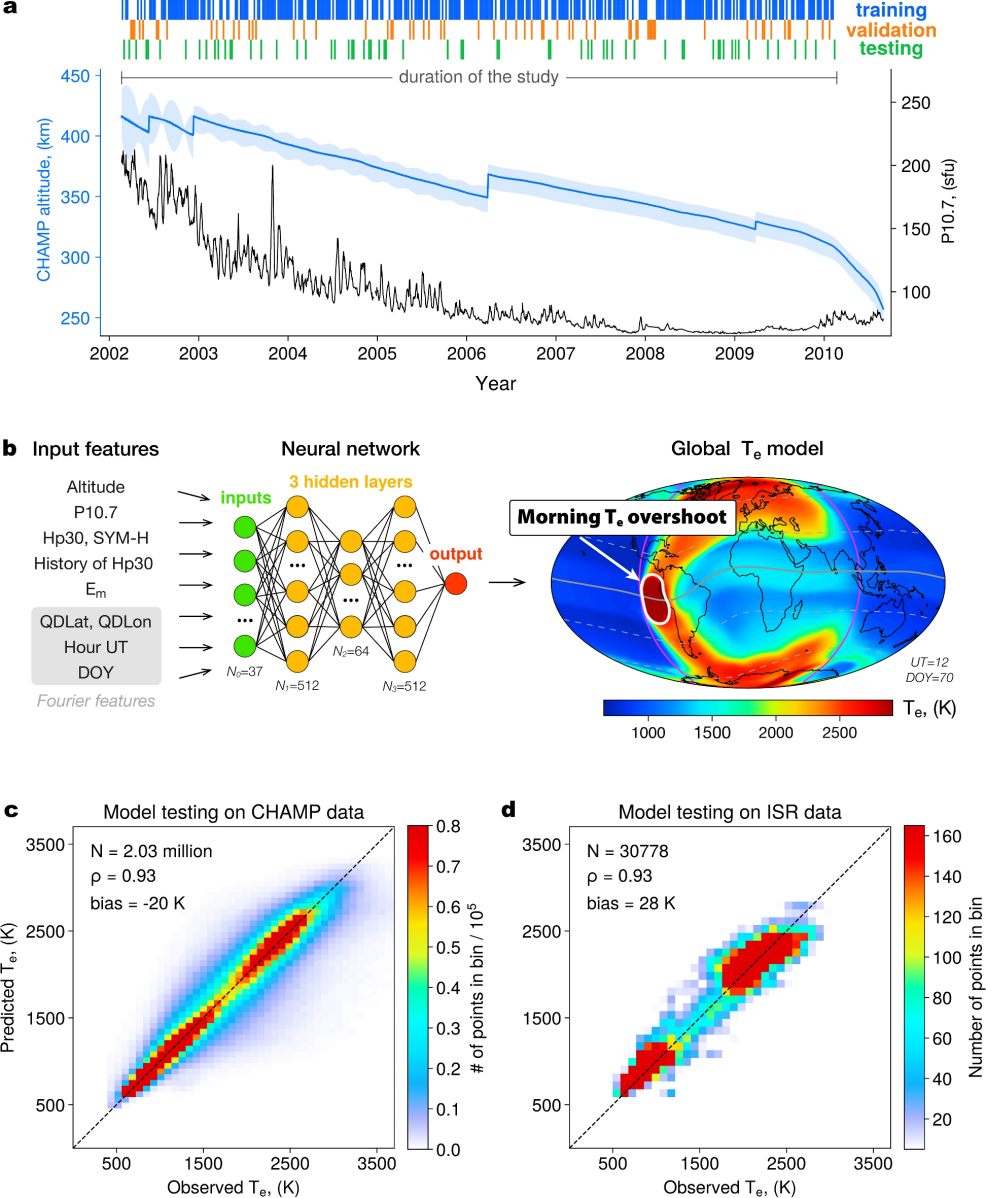
(**a**) Daily mean altitude of the CHAMP satellite (light blue line), and the solar flux index P10.7 for the duration of the study. The daily variations of altitude from maximum to minimum values are shown as a shaded blue area. (**b**) Schematics of the model workflow: the input parameters are supplied to a 3-layer NN to provide a global specification of electron temperature. Magenta line marks the day-night terminator boundary, thick grey line shows the quasi-dipole (QD) equator, and the dashed grey lines indicate QD latitudes of $\pm 30^\circ$ and $\pm 60^\circ$. (**c**, **d**) Model testing on out-of-sample CHAMP and incoherent scatter radar (ISR) data for time periods not used in model training.

Recent advancements in empirical modeling techniques have significantly enhanced our ability to model various ionospheric parameters, such as electron density, total electron content and indices of ionospheric irregularities^15–18^. Systematic efforts to model electron temperatures, however, have remained rather limited^6^. To the best of our knowledge, empirical modeling of electron temperatures during geomagnetic storms has not been attempted. This is partly due to sparsity of global $T_e$ observations available for the model training—a limitation that is even more evident during rare events like geomagnetic storms. Drawing a parallel to weather prediction, this task is akin to reconstructing global weather patterns using one observation at a time. While certainly challenging, this problem can be addressed by using modeling techniques that can learn efficiently from sparse observations. Artificial neural networks have excelled at learning complex non-linear dependencies from sparse point measurements in various space physics applications^17,19–21^ and are used in this study as the basis for developing the electron temperature model. NNs represent one of the techniques that allow creating digital twins, which are virtual representations that describe the behavior and characteristics of real-world physical systems with high fidelity^22^.

The neural network model developed here is trained on global electron temperature observations by the CHAMP mission in 2002–2010 (Fig. 1a). As inputs to the model, we use a combination of features that account for external driving, such as the solar flux index P10.7, smoothed merging solar wind electric field^23^ and geomagnetic indices SYM-H and Hp30 with the time-history of the latter, and features that parametrize the spatial, diurnal and seasonal variations (detailed information about the data and model construction is presented in the Methods section). The input features are supplied to a 3-layer fully connected neural network (Fig. 1b), which outputs electron temperature predictions for given input combinations.

The developed model reproduces CHAMP observations from both the training and validation sets very well, with a Spearman rank correlation of $\sim 0.93-0.94$ and a near-zero bias in the order of $\sim 20$ K (Fig. S1 in the Supporting information). To ensure that the model does not overfit the training data and has a good generalization ability, it is crucial to test it on unseen observations. We first test the model using out-of-sample CHAMP data from time periods not used in model training (Fig. 1c). The metrics evaluated on the test set are almost identical to those for the training and validation sets (see also Fig. S1 in the Supporting Information). This indicates that the model does not show signs of overfitting and has strong generalization capabilities. Additionally, to ensure that the model predictions are in agreement with other electron temperature data sets, we test the model on $T_e$ observations by the incoherent scatter radars (ISRs) (the data description is given in the Methods section). A comparison between the model predictions and ISR measurements (Fig. 1d) indicates a very good agreement between them, with metrics similar to those evaluated on the CHAMP data set. Due to its excellent agreement with out-of-sample observations from both CHAMP and ISRs datasets, the developed model can be considered a digital twin of the ionospheric $T_e$ distribution and can be used to analyze $T_e$ patterns on a global scale.

An example of the global NN run, shown in Fig. 1b, depicts several characteristic features of the global $T_e$ distribution. At low and mid-latitudes, electron temperatures remain low (<1000 K) on the nightside due to the absence of photoionization. $T_e$ values are enhanced along the morning terminator boundary, marked with a magenta line in Fig. 1b. At low and mid-latitudes, electron temperatures increase for a few hours in the early morning (05–08 LT), but quickly fall off around 09 LT and remain relatively stable throughout the day. The global $T_e$ distributions also present a number of patterns at high latitudes, such as a nocturnal $T_e$ increase around the auroral oval, but these features are beyond the scope of the present study and can be a focus of a separate investigation. Of particular interest is the region of the morning $T_e$ overshoot, highlighted with a white oval, where electron temperatures reach $\sim$3000 K and present a global $T_e$ maximum during quiet times.

### Two-stage response of the morning overshoot to geomagnetic storms in NN simulations

**Fig. 2:**
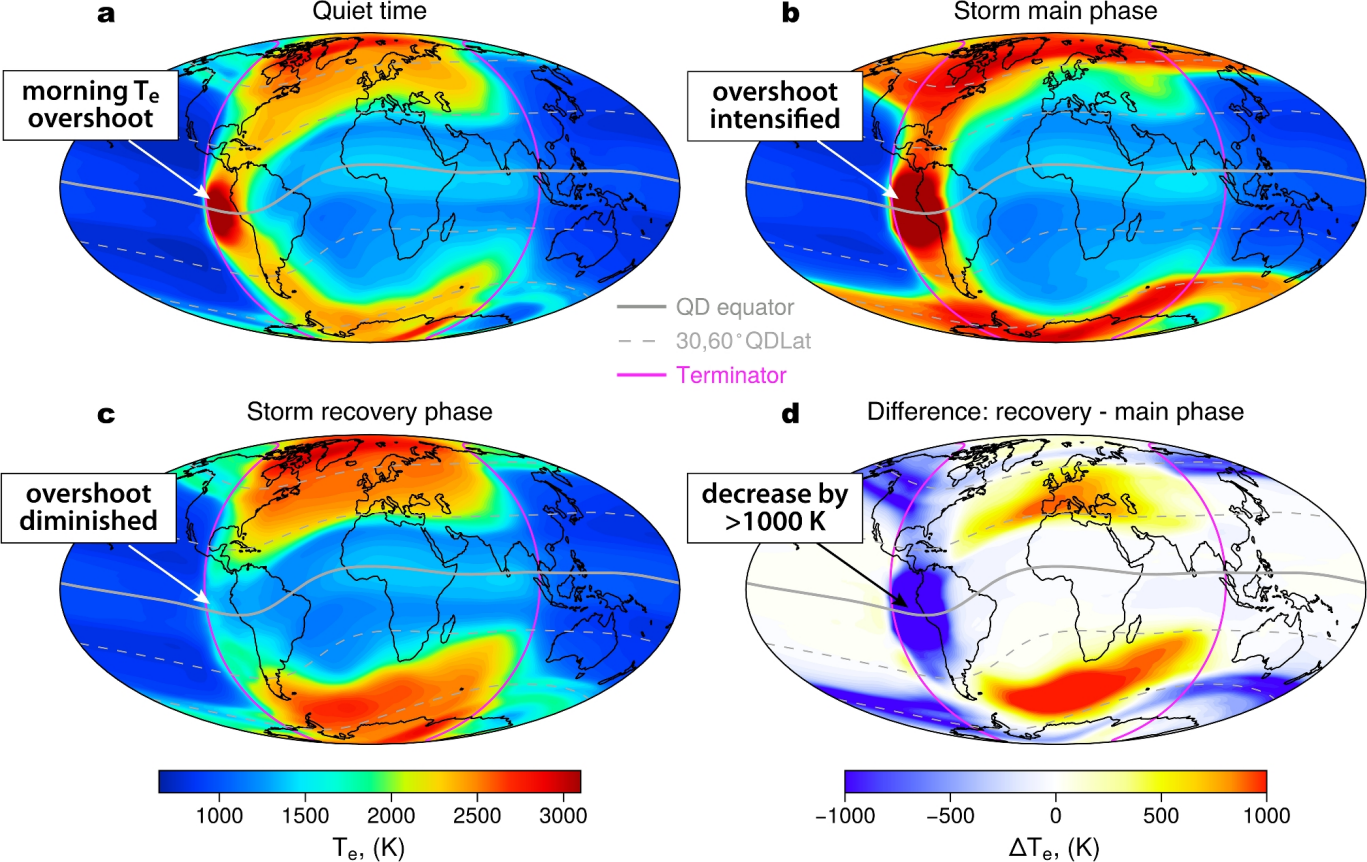
Global NN simulation results, showing electron temperature distributions at 350 km altitude around the Spring equinox (DOY = 87) for different geomagnetic storm phases: Pre-storm (**a**), main phase (**b**) and recovery phase (**c**). Panel (**d**) shows the difference between the recovery and main phases.

To investigate how electron temperatures evolve in the morning overshoot region during different storm phases, we simulated global $T_e$ distributions for pre-storm, main and recovery phase conditions (Fig. 2). In our NN model, the time progression of events is parametrized with a time history of the Hp30 index of up to 12 h (see the Methods section and Supplementary Fig. S2). To simulate the quiet-time $T_e$ distribution (Fig. 2a), we used an Hp30 value of 2 for the preceding 12 h. For the storm main phase, we used an increased instantaneous Hp30 value of 7 (Fig. 2b), which was also used as the time-history input, with a 6-h time-lag, to simulate the $\hbox {T}_e$ distribution during the recovery phase (full inputs are specified in Supplementary Table S2). The results reveal substantial differences in electron temperatures between active and pre-storm conditions (Fig. 2a,b). In particular, the morning $T_e$ overshoot intensifies during the peak activity, with electron temperature values exceeding 3500 K. During the recovery phase, a strong $T_e$ depletion of >1000 K is observed at the equator (Fig. 2d), with a total suppression of the morning overshoot. The values in this region are similar to those at mid-latitudes, and the global $T_e$ maximum is located around the high-latitude cusps instead of the equator.

The NN simulations reveal unexpected storm-time dynamics of the morning electron temperature overshoot, consisting of two distinct phases with initial intensification of the overshoot and the following suppression. This finding needs to be confirmed by satellite observations. In the following 2 subsections, we first present a case study of the geomagnetic storm on 23 April 2003, and later investigate the averaged behavior of $T_e$ in the overshoot region for different geomagnetic activity levels and time-lags.

### Case study: morning $T_e$ overshoot during the 23 April 2003 storm

**Fig. 3:**
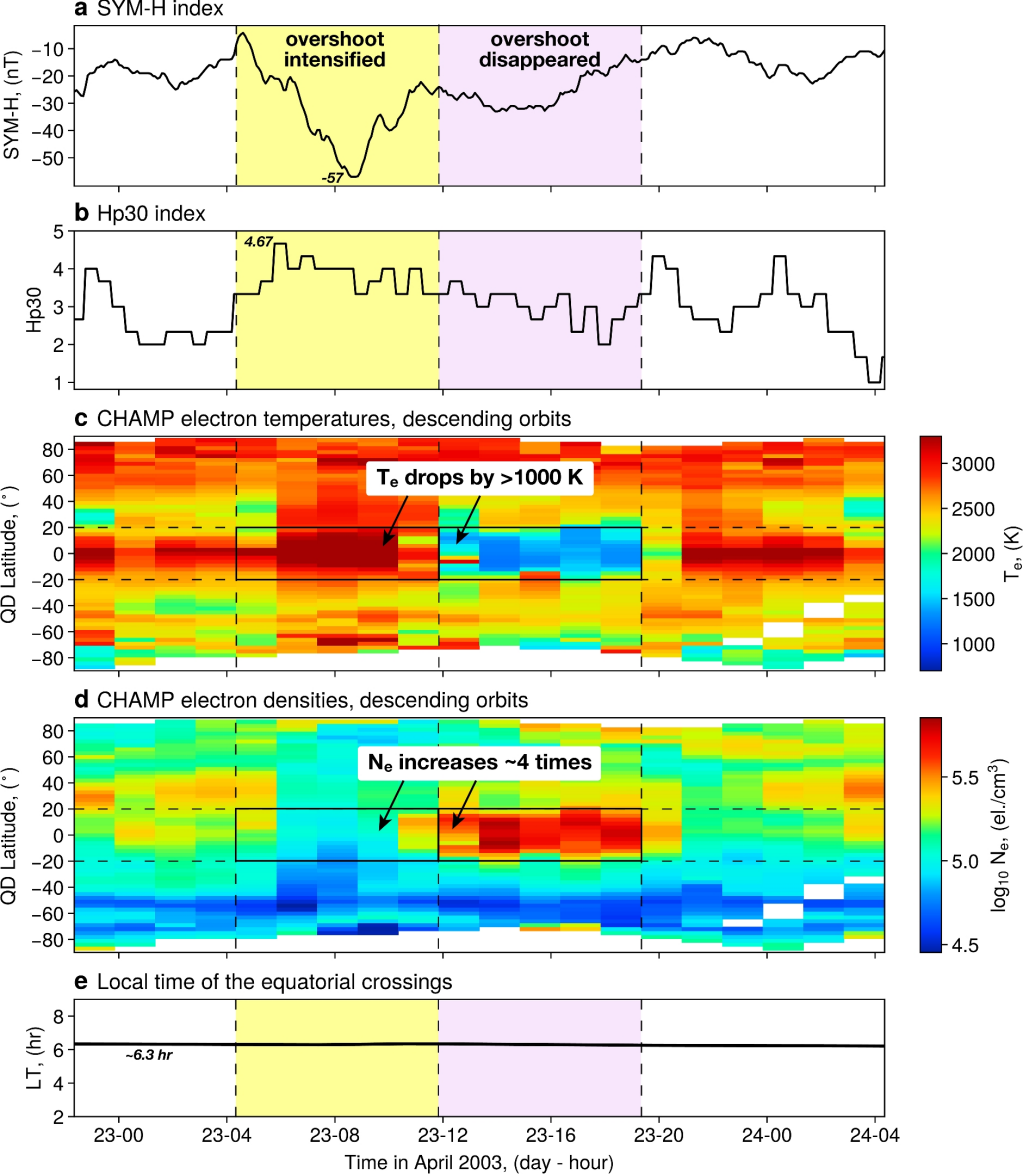
A case study of electron temperature and density response to the 23 April 2003 geomagnetic storm. Panels (**a**) and (**b**) show SYM-H and Hp30 geomagnetic indices for the duration of the event. Panels (**c**) and (**d**) show CHAMP observations of electron temperature and density, respectively for descending orbits, as functions of the quasi-dipole latitude and time. The consecutive bins are separated in time by $\sim$1.5 h. Panel (**e**) shows local time of the equatorial crossings by CHAMP. The satellite crossed the equator in its descending orbits at a fixed LT of 6.3 h throughout the event.

Figure 3 shows electron temperature and density observations by CHAMP during the geomagnetic storm on 23 April 2003. This event corresponded to the maximum Hp30 value of 4.67 and a SYM-H index minimum of − 57 nT (Fig. 3a,b). Electron temperatures and densities observed by CHAMP, plotted as functions of quasi-dipole latitude (QDLat) and time, are shown in panels (c) and (d), respectively.

During the event, the morning $T_e$ overshoot displayed a two-phase change consistent with the pattern observed in the NN simulations (Fig. 2). Electron temperatures in the equatorial region increased from $\sim 2600$ K to above 3200 K around the time of Hp30 maximum (05 UT), and continued to rise over the following $\sim 4$ h. This period corresponded to depleted values of electron density (Fig. 3d). Approximately 4–5 h after the activity peak, electron temperatures started to decrease, with a drop of $>1000$ K over a period of $\sim 3$ h. This coincided with a fast build-up of electron density by a factor of $\sim 4$. It should be noted that electron temperatures around the equator during this period were lower than at mid-latitudes, showing a complete suppression of the $T_e$ overshoot. At mid-latitudes, a $T_e$ depletion was observed in the Northern hemisphere (a decrease of about 200 K), but only slight changes occurred in the Southern hemisphere. The suppression of the morning overshoot lasted approximately 7.5 h until the next increase in Hp30 around 20 UT.

### Statistical analysis of morning $T_e$ overshoot response to geomagnetic activity

The lagged two-stage response of the $T_e$ overshoot to geomagnetic activity, observed in the neural network results and the case study of the 23 April 2003 event, can be analyzed statistically for different geomagnetic activity levels and timelags. Using the entire CHAMP data set, we select geomagnetic storms where maximum Hp30 values over the period of $\pm 24$ h corresponded to 4.67, 5, 5.3, and 5.67 and above. Due to the fact that the morning overshoot represents a relatively small feature on spatial scale, few CHAMP measurements in the overshoot region can be found for strong geomagnetic storms with Hp > 6, and in order to have more representative statistics, events with Hp above 5.67 are binned together in a “high activity” bin. Additionally, when selecting events for the averaging, we use an overlap in Hp30 of $\pm 0.33$ in order to provide a smoother transition between the bins. In order to remove the influence of repeating storms, we put additional criteria on the event selection: (1) the average Hp30 value from 24 up to 6 h prior to the storm peak must be below 3, and (2) the maximum Hp30 level from 6 to 24 h after the storm peak should be below 4.66. We consider times of Hp30 maxima as the 0-th epochs for each storm, and calculate the average values of electron temperature and density for time-lags from $-15$ to + 18 h. This allows us to track the progression of storm-time changes in both $T_e$ and $N_e$ for geomagnetic storms of different strengths. This method is different from the commonly used superposed epoch analysis where all storms exceeding a certain Hp30/SYM-H threshold are binned together^24^. Our results show that the response of $T_e$ and $N_e$ varies depending on the storm strength, and therefore averaging values for all storms together would not be appropriate.

**Fig. 4:**
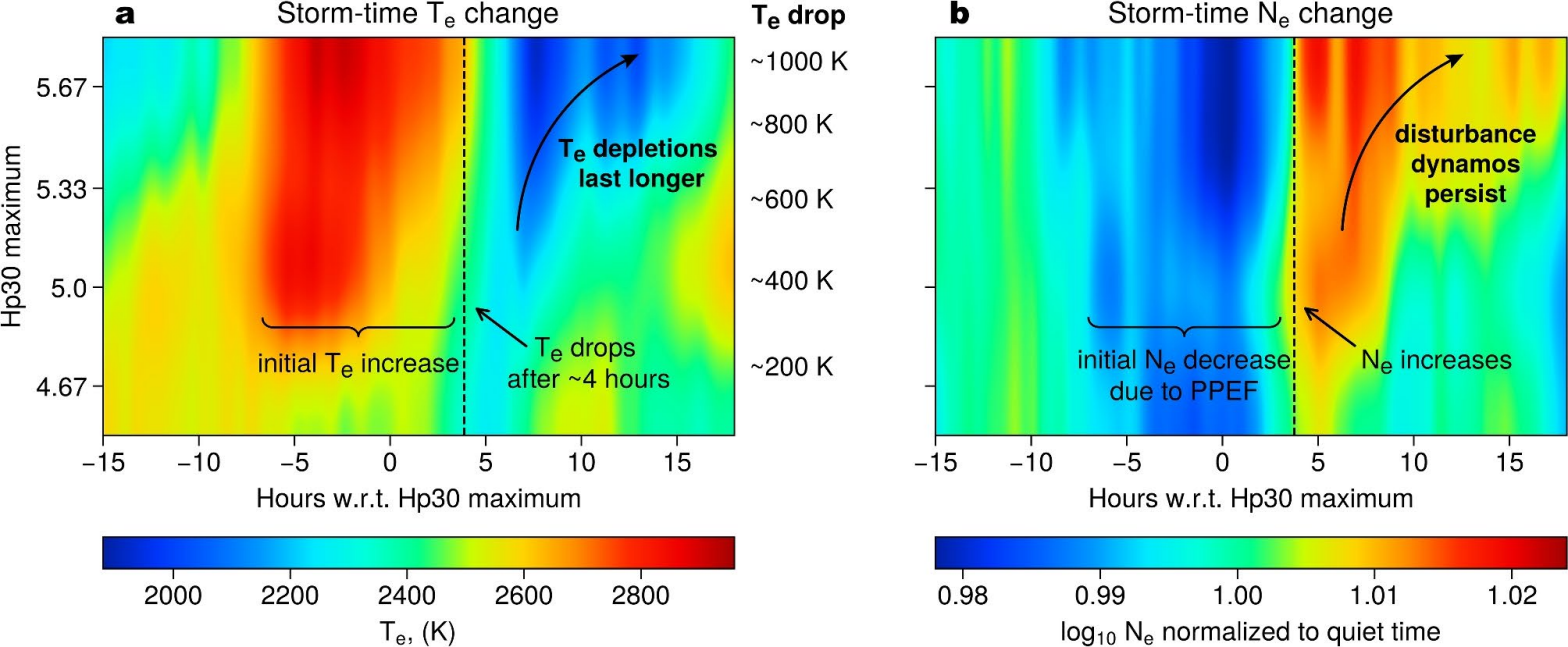
Lagged response of electron temperature and density in the morning overshoot region (05–07 h LT and |QDLat|$<10^\circ$) to geomagnetic activity. (**a**) Average values of electron temperature, color-coded as a function of the Hp30 geomagnetic index and the time-delay since a given Hp value is reached. (**b**) Storm-time variations of electron densities, normalized with respect to quiet times ($\log _{10}N_e$/$\log _{10}N_e^{quiet}$).

Statistical results in Fig. 4 also demonstrate that electron temperatures and densities in the overshoot region react to geomagnetic activity in two phases. In the first phase, $T_e$ increases with geomagnetic activity and persists for about 4 h after the Hp30 maximum. Then, a sudden drop of $T_e$ marks the onset of the second phase. Notably, the timing of the initial $T_e$ depletion is roughly consistent across storms of all intensities, with electron temperatures dropping below their quiet-time values. The later changes, however, do depend on the Hp30 level. For mild and moderate events (Hp30$\le$5), electron temperatures return to approximately pre-storm levels within 3–4 h after the initial depletion. In contrast, for strong storms (Hp30 > 5), electron temperature continues to decline over longer periods of time. The duration of these prolonged depletions increases with the storm strength. Furthermore, the magnitude of the overall temperature decrease also depends on the maximum Hp30 level: moderate events show depletions of 200–400 K, while stronger events correspond to overall $T_e$ drops of >1000 K on average.

The lagged depletions of electron temperature after geomagnetic storm’s main phase coincide with increases of electron densities (Fig. 4b). In particular, electron densities start to build up around 3–4 h after the Hp30 maximum (marked with a vertical dashed line in Fig. 4b). Following this initial increase, $N_e$ tends to stay at elevated levels for several hours into the recovery phase, and the duration of this increase depends on the Hp30 level. For instance, for moderate events (Hp30 < 5) the $N_e$ is enhanced for 2–3 h, while for stronger events (Hp30 > 5.3) the electron density remains at elevated levels compared to the quiet time for up to 12–15 h. We note that electron density values can vary by several orders of magnitude in the equatorial region, and therefore in Fig. 4b the storm-time $N_e$ variation is normalized with respect to quiet time (as $\log _{10}N_e$ / $\log _{10}N_e^{quiet}$), and the unnormalized values are provided in Supplementary Fig. S3. In contrast, electron temperature values in Fig. 4a are shown without normalization, as this study primarily focuses on the magnitudes of $T_e$ drops during geomagnetic storms.

## Discussion and outlook

In this study, we developed a NN model of electron temperature in the F-region of the ionosphere. Due to its excellent performance on independent observations, the model can be considered a digital twin of the global $T_e$ distribution at altitudes of 320–400 km. Our model uncovered an unexpected behavior of the morning $T_e$ overshoot during geomagnetic storms, consisting of two distinct phases. At first, $T_e$ increases during the main phase of the storm. This is followed by an intense cooling of the overshoot region and a near-total suppression of the morning $T_e$ overshoot in the recovery phase.

Electron temperatures are governed by several mechanisms that can be summarized in the electron energy conservation equation^3^:

1 $$\begin{aligned} \dfrac{3}{2} N_e k \dfrac{\partial T_e}{\partial t} = \sin ^2 I \dfrac{\partial }{\partial z} \left( K^e \dfrac{\partial T_e}{\partial z} \right) + \sum Q_e - \sum L_e, \end{aligned}$$

where $\sum Q_e$ and $\sum L_e$ represent sums of all the cooling and heating rates, respectively, $K^e$ is the electron thermal conductivity in the limit of zero current, *I* is the dip-angle, *k* is the Boltzmann constant, and *z* is altitude. The first term on the right hand side represents the electron thermal conduction. It is negligible around the equator where the magnetic field lines are nearly horizontal ($\sin I = 0$). The terms related to field-aligned currents (FACs) are omitted from Eq. (1) due to their minor impact at the equator. Therefore, the behavior of electron temperature around the morning overshoot can be explained by a balance between the heating and cooling mechanisms. It has been established that the heating rates are linearly proportional to electron density, while the cooling terms scale with the square of $N_e$^3,6^. With increasing electron density, the cooling rapidly overtakes the heating and would prevent the development of the $T_e$ overshoot. As a consequence, there is a well-established anticorrelation of electron density and temperature around the morning overshoot region^25–27^. The storm-time $T_e$ variations are therefore mainly controlled by the storm-induced modulations of electron density.

Increasing geomagnetic activity triggers the first stage of the storm-related changes in the morning overshoot region. It consists of the initial increase of $T_e$ and depletion of $N_e$ (Figs. 3 and 4). We interpret these initial changes as a consequence of the prompt penetration electric fields altering the direction of the vertical plasma drift at the equator. During geomagnetically quiet times, the pre-dawn electric field in the equatorial region is directed westward^28^, leading to the downward **E × B** drift that reduces electron density and presents a necessary condition for the morning $T_e$ overshoot to develop^7^. Slight magnetospheric disturbances, which map to high latitudes, are usually shielded from reaching low and mid-latitudes by the Region-2 FACs^29^. During geomagnetic storms, however, fast and intense fluctuations of the magnetospheric electric fields are strong enough to overcome this shielding and can penetrate rapidly towards the equator on timescales of about 1–2 h^30^. It has been shown that around sunrise (05–06 h LT), the PPEFs enhance the westward component of the equatorial electric field^29,30^. This configuration leads to an intensification of the downward **E × B** drift, which pushes the ionized particles into regions of denser atmosphere with high recombination rates and thus reduces electron density below the quiet-time levels^31^. The newly ionized photoelectrons at sunrise exchange their energy with fewer ambient electrons, resulting in a higher energy share per electron^6^ and enhanced electron temperatures in the morning overshoot region.

The second phase begins around 4–5 h after the geomagnetic activity peak. It is manifested by an initial depletion of electron temperature and prolonged subsequent cooling (Fig. 4). These $T_e$ changes are also anti-correlated with electron density (Figs. 3 and 4). We attribute the development of the cooling phase to the action of the storm-induced disturbance dynamo electric fields^32,33^. After the input into high latitudes in the form of particle precipitation and Joule heating, temperatures of neutral atmosphere at high latitudes are increased, which creates a pressure gradient with respect to lower latitudes, driving strong equatorward thermospheric winds^32^. Due to the inertia of neutral air, it takes around 3–5 h to setup the neutral winds after the high-latitude disturbances, but once generated they can persist for over a day^33^. These winds generate the DDEF that is anti-parallel to the quiet-time equatorial electric field. At the equator, the disturbance dynamo has the strongest effect precisely around the overshoot region (around 06 LT) and generates eastward electric field^24,29^. This configuration flips the **E × B** drift from downward to upward, lifting more electrons to the topside ionosphere and increasing $N_e$ (Fig. 4b). As a consequence, the enhanced cooling leads to a depletion of $T_e$ (Fig. 4).

The overall $T_e$ depletions in the second (cooling) stage are more pronounced for stronger geomagnetic storms. Furthermore, the duration of the prolonged cooling is also dependent on the storm strength. For moderate events corresponding to Hp30$\le 5$, the cooling stage lasts for around 3–4 h, and electron temperatures rebound to the quiet-time levels. For stronger events, however, $T_e$ remains depleted for up to 12–15 h after the maximum activity levels are reached. These periods correspond to gradual increases of electron density (Fig. 4). These changes agree well with the behavior of disturbance dynamos. Fejer and Scherliess^29^ showed that for strong geomagnetic storms, the eastward disturbance electric fields around the equator at 06 LT persist well into the recovery phase, lasting for more than 24 h. Their study demonstrated that the strength of the resulting upward **E × B** drift is directly proportional to the high-latitude input during disturbed times. Our findings agree well with these results: the persisting upward **E × B** drifts allow electron density to gradually build up over time. This creates the necessary conditions for extreme depletions of electron temperatures by $>1000$ K on average for strong geomagnetic storms (Hp30 > 5).

The digital twin electron temperature model developed in this study can have broad applications in ionospheric research. Firstly, the model revealed a new physical pattern in the equatorial ionosphere, showing that the morning $T_e$ overshoot reacts to geomagnetic activity in two distinct phases. This finding led to a revised understanding of this well-studied ionospheric feature. Due to the simplicity of the model, requiring only a few external parameters, it can be easily applied to investigate storm-time $T_e$ variations in other regions, including high latitudes. Furthermore, the model can be adapted for real-time operations. Since $T_e$ typically increases prior to the ion and neutral temperatures^2,3^, it can serve as an early indicator of various processes related to magnetosphere-ionosphere-thermosphere coupling, including during severe storms. It should be noted that while a simple two-stage explanation of the morning $T_e$ overshoot dynamics provides a consistent view of the region’s behavior during active times, each geomagnetic storm presents a unique chain of events that may not always align with this simplified explanation. Ultimately, the redistribution of electron density and the corresponding changes in $T_e$ are determined by the equatorial electrodynamics, which is influenced by many contributions, not all of them described by the Hp30 index. Therefore, our study also shows the need for new measurements in the topside ionosphere that would cover a range of parameters, including electron temperatures, densities and plasma drifts and would allow studying their drivers through coordinated observations.

## Conclusions

In this study, we developed a digital twin model of the ionospheric electron temperatures based on 8 years of global observations by the CHAMP mission. Our modeling results have uncovered a new physical pattern in the equatorial ionosphere, namely, the two-phase change of the morning electron temperature overshoot during geomagnetic storms. This response consists of an initial $T_e$ enhancement during the main phase, followed by a pronounced cooling and suppression of the overshoot in the recovery phase. Confirming these results by observations, we propose that these changes are driven by the redistribution of the electron densities in response to storm-induced changes in the equatorial electric fields. The initial intensification of the overshoot is consistent with the prompt penetration electric fields acting within the first few hours of the storm, while the subsequent disappearance of the overshoot is attributed to the development of disturbance wind dynamos. These findings provide a revised understanding of the storm-time behavior of the morning $T_e$ overshoot, which is one of the most widely studied ionospheric features. Our results demonstrate the potential of advanced empirical models, particularly those based on artificial intelligence, to reveal previously unrecognized physical patterns even for the most commonly studied phenomena. Digital twin models offer unique capabilities to construct global views of complex systems under varying conditions, not achievable from sparse point measurements, and can lead to discovering new physical mechanisms in the near-Earth space environment and across other fields of study.

## Methods

### Data set

This study is based on electron temperature and density observations by the Planar Langmuir Probes (PLPs) aboard the CHAMP mission^14^. The CHAMP satellite was launched in mid-2000 into near-polar orbit with an inclination of $87.25^\circ$^14,34^. The initial altitude of CHAMP was $\sim$460 km, slowly decaying to below 300 km in 2010. CHAMP covered different local time sectors, providing a complete LT-coverage over the period of $\sim 130$ days^34^. The PLP instrument on CHAMP represented a golden rectangular plate with a 106$\times$156 mm area, mounted at the lower front panel of the satellite, and operated in a voltage sweep mode^6,34^. PLP took measurements every 15 seconds, by tracking the spacecraft potential for 14 s and then sweeping the voltage to obtain electron density and temperature^35^. CHAMP traveled with a velocity of $\sim$7.6 km/s, and therefore the PLP data have a horizontal resolution of roughly 115 km^34^. CHAMP electron temperature observations are available for the period between the 19 February 2002 and 22 February 2010^36^. We use $T_e$ observations in the range from 400 to 4500 K, and select measurements where the corresponding electron densities were above $2 \times 10^4$ $\hbox {cm}^{-3}$, to avoid potential biases of Langmuir probes in regions of extremely low plasma densities (typically around the mid-latitude trough)^37^. The CHAMP electron temperature observations were calibrated by the incoherent scatter radars^34^ and present a practically calibration-free dataset of electron temperatures that can be used for empirical modeling.

To parametrize the external driving of the electron temperature variability, we utilize several geomagnetic and solar activity parameters. As a proxy of solar activity, we use the solar flux index P10.7, which represents a smoother version of the 10.7 cm radio flux (for details, see e.g., Bilitza and Xiong^38^). We use the Hp30 index^39^, which is a proxy of the geomagnetic activity on planetary scale, and is calculated similarly to the widely used Kp index but with a higher resolution of 30 min. An additional advantage of the Hp30 is that it is not saturated at 9.0 and has been shown to exceed 12 units during geomagnetically active conditions. Furthermore, we use the SYM-H index which is a proxy of the geomagnetic storm intensity. To account for the coupling between the solar wind and ionosphere-magnetosphere system, we use the merging electric field^23^, calculated as follows:

2 $$\begin{aligned} E^\prime _m = \dfrac{1}{3000}V_{sw}^{4/3} \left( \sqrt{B_y^2 + B_z^2} \right) ^{2/3} \sin ^{8/3} \left( \dfrac{\theta }{2} \right) , \end{aligned}$$

where $V_{sw}$ is the solar wind velocity (in km/s), $B_y$ and $B_z$ represent the interplanetary magnetic field (IMF) components in GSM coordinates (measured in nT), and $\theta = \tan ^{-1} (B_y/B_z)$ is the clock angle of the IMF. The solar wind data, obtained from the OMNIWeb database, are already propagated to the Earth’s bow shock. $E^\prime _m$ has units of mV/m and is comparable in magnitude with the solar wind electric field^40^. It should be noted that the response of the polar ionosphere to changes in the solar wind input are not immediate, and are lagged by 1–2 h^30,33,41^, and furthermore not every small fluctuation in the solar wind causes changes in the ionosphere. To take this timelag into account, we use the exponential-folding smoothing of the merging electric field as follows:

3 $$\begin{aligned} E_m (t^\prime ) = \dfrac{\int _{t_1}^{t} E^\prime _m(t^\prime ) e^{(t^\prime -t)/\tau }dt^\prime }{\int _{t_1}^{t} e^{(t^\prime -t)/\tau }dt^\prime }, \end{aligned}$$

where $t^\prime$ is the time in question, $t_1$ = 3 h is the window for smoothing and $\tau$ is the exp-folding time equal to 0.5 h. We apply similar smoothing to Hp30 and SYM-H indices, choosing $t_1$ and $\tau$ to be 6 and 1 h, respectively. In the case of the Hp30 index, the smoothed values are calculated by linearly interpolating the ap30 index to a 5-min cadence, applying the exp-folded smoothing to those values and then converting them to the Hp-scale using a semi-logarithmic look-up table.

To parametrize the temporal evolution of electron temperatures during geomagnetic storms, we include the time-history of the Hp30 index to the model inputs. We consider the time-lags of 1, 3, 6, 9, 12, 15, 18 and 24 h. We first train a model using Hp30 values at all timelags and evaluate the so-called permutation feature importances (Fig. S2 in the Supporting Information). The importance score for each variable is calculated as the increase in prediction error on the validation set resulting from randomly shuffling the values in the corresponding input column. Due to the fact that the Hp30 values with lags beyond 12 h have very low importance scores, we used the time-history of Hp30 of up to 12 h. Incorporating the time-history of external drivers (in our case, Hp30) allows the developed model to predict electron temperature values based on the information about the preceding geomagnetic activity conditions. This is crucial for geomagnetically active times, since storms of different strengths typically correspond to different ionospheric responses^42^.

We test our NN global $T_e$ model against observations collected by several ISRs, which are considered to be the “gold standard” of the topside ionospheric observations. ISRs are ground-based facilities that exploit the Thomson backscatter from ionospheric electrons to obtain a number of ionospheric parameters, including $N_e$ and $T_e$, over a range of altitudes covering the entire ionosphere^43^. We select $T_e$ observations collected by three ISRs: Jicamarca ($12.0^\circ$ S, $76.8^\circ$ W; QDLat $0.2^\circ$ N), Arecibo ($18.2^\circ$ N, $66.4^\circ$ W; QDLat. $27.0^\circ$ N), and Millstone Hill ($42.6^\circ$ N, $71.5^\circ$ W; QDLat $51.8^\circ$ N). This allows us to test the model under different conditions imposed by the large latitudinal variations of the ionosphere, from the geomagnetic equator (Jicamarca ISR), to the low latitudes (Arecibo ISR), to the midlatitudes/sub-auroral latitudes (Millstone Hill ISR). The ISRs dataset covers the time periods not used in model training (test periods, see Fig. 1a and the following section on machine learning methodology). However, since ISRs do not operate continuously over time, ISR observations are not available for all the test time periods, and thus represent a sub-dataset of the test periods shown in Fig. 1a. We consider ISRs observations in the height range between 310 and 400 km of altitude, similar to the CHAMP altitude coverage used for training, in order not to extrapolate the model output. To filter out noisy $T_e$ measurements, we consider only the observations complying with the following criteria: for Jicamarca and Arecibo, we consider data with DTe < 200 K, DTi < 200 K and DNe < 1e10 $\hbox {m}^{-3}$, where DTe, DTi, and DNe stand for the measurement errors associated to electron temperature, ion temperature, and electron density observations, respectively; for Millstone Hill, we consider data with a signal-to-noise ratio > 0.75 and exclude irregular readings from 11/11/2003. For all ISRs, we select the $T_e$ data between 400 and 4500 K, to avoid unphysical values in the F-region ionosphere. After filtering, the ISRs dataset reduced to around 30 thousand $T_e$ observations. The NN $T_e$ model is run for the same times, locations, and solar and geomagnetic activity conditions of the selected ISRs observations. ISRs data are downloaded from the Madrigal database, freely accessible at http://cedar.openmadrigal.org.

### Machine learning methodology

The near-Earth space environment represents a region traditionally undersampled by in-situ observations. Due to the paucity of the available observations, one should select modeling techniques which can learn effectively and generalize from sparse measurements. Artificial neural networks have been shown to excel at this task for space physics applications^17,19–21^. Therefore, in this work we use NNs to develop the electron temperature model using the global data set provided by the CHAMP mission. We use the multilayer perceptrons (MLPs), which represent a type of fully connected NNs. MLPs consist of series of interconnected nodes, organized into layers. Each node has an associated trainable weight and bias, and the process of MLP training involves optimizing these weights and biases, typically using gradient descent algorithms with backpropagation of errors^44^. MLPs can learn highly complex mappings between the input and output variables, due to the non-linearity introduced by the activation functions.

We develop an MLP model to predict a numeric output which can be represented as a continuous variable, and therefore address a regression problem. To parametrize the spatial distribution of electron temperature, we add altitude and quasi-dipole (QD) latitude and longitude, as well as hour of the day and day of the year to account for daily and seasonal variations, respectively. We employ the Fourier features technique to replace the cyclic features, such as the QDLat and QDLon, hour UT and DOY with their sine and cosine transformations up to a given degree^45^. This method has been originally applied to image regression problems and showed a significant improvement achieved by replacing the pixel coordinates with the Fourier transformed values. In relation to space physics problems, this method has also shown a very good performance for ionospheric modeling^17^, and is therefore also applied here. We empirically selected the Fourier features of QDLat up to degree 4 and those of QDLon, UT and DOY up to degree 3. The input variables that account for external driving are described above and contain a combination of solar flux index P10.7, solar wind merging electric field and geomagnetic indices Hp30 and SYM-H (with a time-derivative).

MLP models have large numbers of parameters, frequently in the range of hundreds of thousands of trainable links between neurons. This makes MLPs prone to overfitting the training set without generalizing well onto the unseen observations. Thus, it is crucial to check model performance not only on data that were used to fit the model, but also on validation data, especially when selecting the optimal hyperparameter combinations. Additionally, after the models have been tuned and trained, it is necessary to check the performance on a fully independent set of data which has not been used in the model construction, which is referred to as the testing set. There are several strategies which can be used for data splitting. Random splitting, where the data points are divided into the three subsets randomly, can lead to data leakage when working with time series and spatio-temporal problems and should be avoided^17,46^. Another approach is to use the K-fold cross validation, where the entire data set unsed for model development is divided into K parts and the model is retrained K times each time withholding one part for the validation and using K − 1 parts for training. This approach is effective in preventing the data leakage but leads to significantly higher computational times, with the complexity growing linearly with K. In this study, we apply a slightly different approach, which we refer to as block randomization. We divide the data into weekly intervals, and split those continuous data blocks into the 3 sets randomly. The weekly duration was chosen as it is much longer than the ionospheric memory, but short enough so that the satellite orbit does not drift significantly in local time between the training and test sets^47^. This technique has been applied to several space physics problems and has been shown to provide good performance while reducing the computational complexity^17,20^. After evaluating the model quality on the test set, the NN is retrained on the entirety of the CHAMP data set, in order to provide a better coverage of different conditions sampled by the satellite in the finalized model.

In this study, we build the MLP models using the Keras Python library^48^ with JAX backend^49^. We optimize the hyperparameters of the networks, namely the number of neurons in the hidden layers, initial learning rate, and the batch size using the tree-structured Parzen estimator algorithm as implemented in Optuna Python library^50^. The hyperparameters search domains and the optimized values can be found in the Supporting Information, Table S1.

## Supplementary Information

Supplementary Information.

## Data Availability

All data used in this study are publicly available. The solar (wind) data and the SYM-H index were obtained from the OMNIWeb database (https://omniweb.gsfc.nasa.gov/). CHAMP observations were obtained from the Data Services archive of GFZ Potsdam (https://doi.org/10.5880/GFZ.2.3.2019.007). The Hp30 index is also provided by GFZ Potsdam (https://kp.gfz-potsdam.de/hp30-hp60). Jicamarca, Arecibo, and Millstone Hill ISRs data are available via the public access Madrigal portal at http://cedar.openmadrigal.org. The data and model files, as well as the source codes are provided through the open access Zenodo repository: https://doi.org/10.5281/zenodo.14770872.
