# Supplementary material for: Extreme two-phase change of ionospheric electron temperature overshoot during geomagnetic storms
Source: Sci Rep. 2025 Feb 11;15:5043. doi: 10.1038/s41598-025-89602-z (PMC11814276; doi:10.1038/s41598-025-89602-z)
Supplement: Supplementary file 1 — Supplementary Information. [file 41598_2025_89602_MOESM1_ESM.pdf]

Supplementary Information for "Extreme two-phase change of ionospheric electron temperature overshoot during geomagnetic storms"

Artem Smirnov[1,2,*], Yuri Shprits[2,3], Hermann Lühr[2], Alessio Pignalberi[4], Elena Kronberg[1], Fabricio Prol[5,6], and Chao Xiong[7]

[1]Department of Earth and Environmental Sciences, Ludwig Maximilian University of Munich (LMU), Munich, Germany
[2]GFZ Helmholtz Centre for Geosciences, Potsdam, Germany
[3]Department of Earth, Planetary and Space Sciences, University of California Los Angeles (UCLA), CA, USA
[4]Instituto Nazionale di Geofisica e Vulcanologia (INGV), Rome, Italy
[5]Finnish Geospatial Research Institute (FGI), National Land Survey of Finland (NLS), Espoo, Finland
[6]School of Technology and Innovation, University of Vaasa, Vaasa, Finland
[7]Department of Space Physics, College of Electronic Information, Wuhan University, Wuhan, China
*artem.smirnov@gfz.de

Table S1. Neural network (NN) hyperparameters, their search domains and the optimized values for the final model. The dropout rate and the magnitude of gaussian noise were fixed at 0.05 and 0.126 as these values of the regularization were found to provide a good model performance while avoiding the oscillatory effects on the model output. The *Nadam* optimizer, which was used for the model training, performs iterative adjustments of learning rates individually for each of the parameters; the corresponding learning rates are provided with the models by the link in the Acknowledgements section.

Hyperparameter	Search range	Optimized value
# neurons in the 1st layer	32–512	512
# neurons in the 2nd layer	32–512	64
# neurons in the 3rd layer	32–512	512
batch size	32–512	128

Table S2. External input parameters for synthetic model runs in Figure 2.

Input parameter	Quiet time	Storm main phase	Storm recovery phase
Hp30	2	7	3
Hp30 1h ago	2	4.67	3
Hp30 2h ago	2	3.67	3.67
Hp30 3h ago	2	3	4.33
Hp30 6h ago	2	2	7
Hp30 9h ago	2	2	4.67
Hp30 12h ago	2	2	3.67
SYM-H	-10	-80	-30
E_m	1	5	2
P10.7	100	100	100

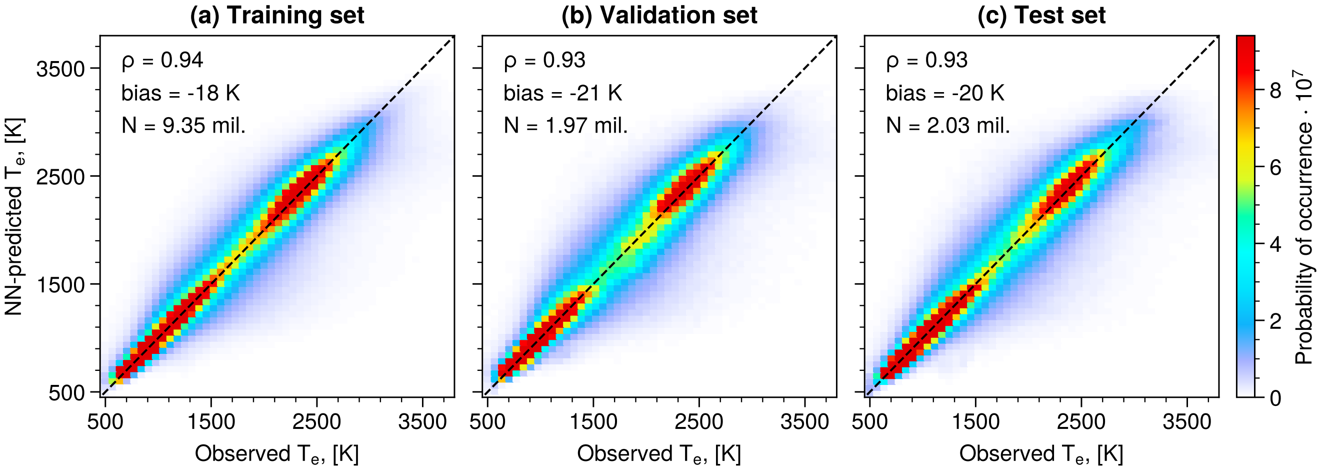

Figure S1. 2D histograms of observed versus predicted electron temperature for the CHAMP data. Panels (a), (b) and (c) show results for the training, validation and test sets, respectively. Each subplot gives values of the Spearman rank correlation (ρ), average bias and the number of points (N). The one-to-one correspondence lines are shown as dotted black lines. One can see that the NN model reproduces electron temperatures well on all three sets, with metrics values comparable on the unseen data from the test subset and those used in model training (panels a-c), which indicates a good generalization ability of the model and minimal overfitting.

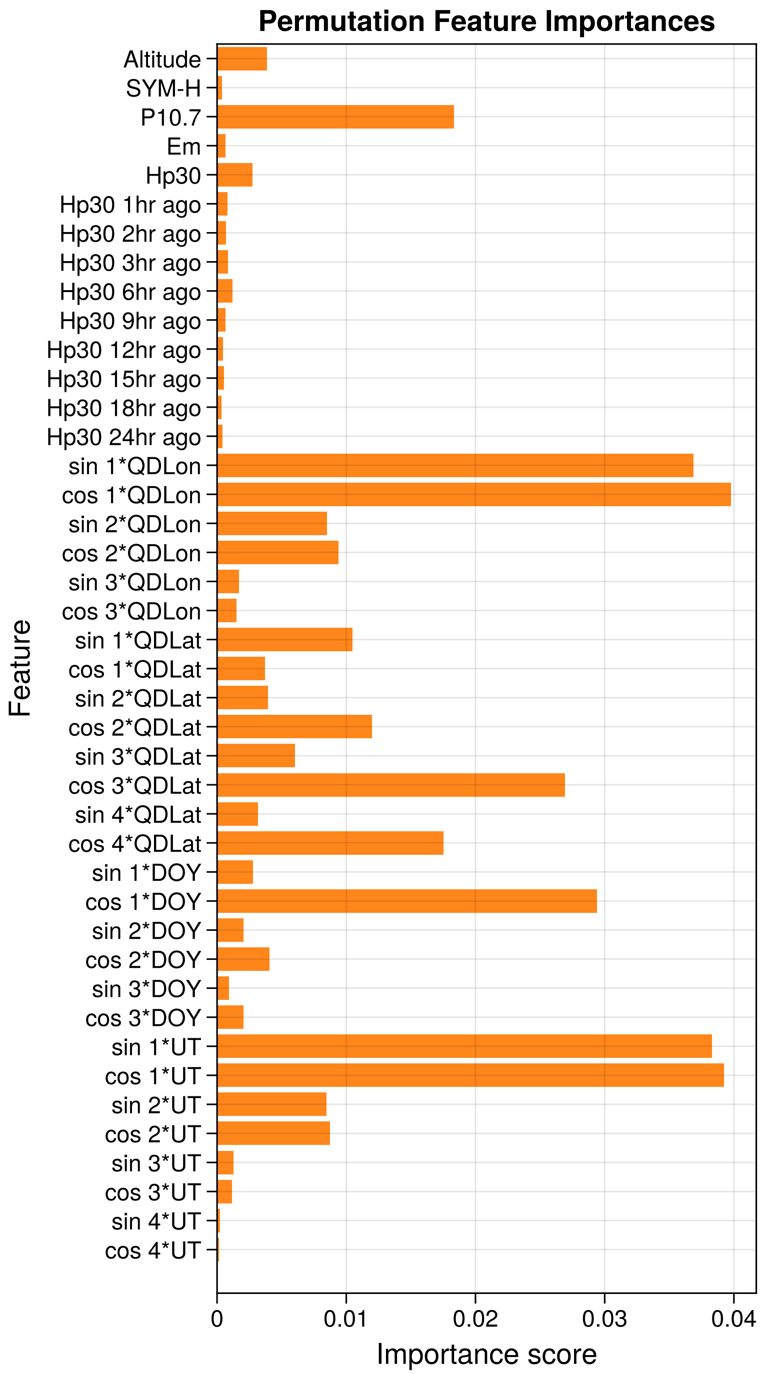

Figure S2. Permutation feature importances on the validation set (their calculation procedure is described in the Methods section). The fourth harmonic of UT has very low importance score and was therefore removed from the inputs. Furthermore, time-history of the Hp30 index has relatively low importance beyond the 12-hour time-lag. Therefore, the final model is trained using time-history of Hp30 of up to 12 hours.

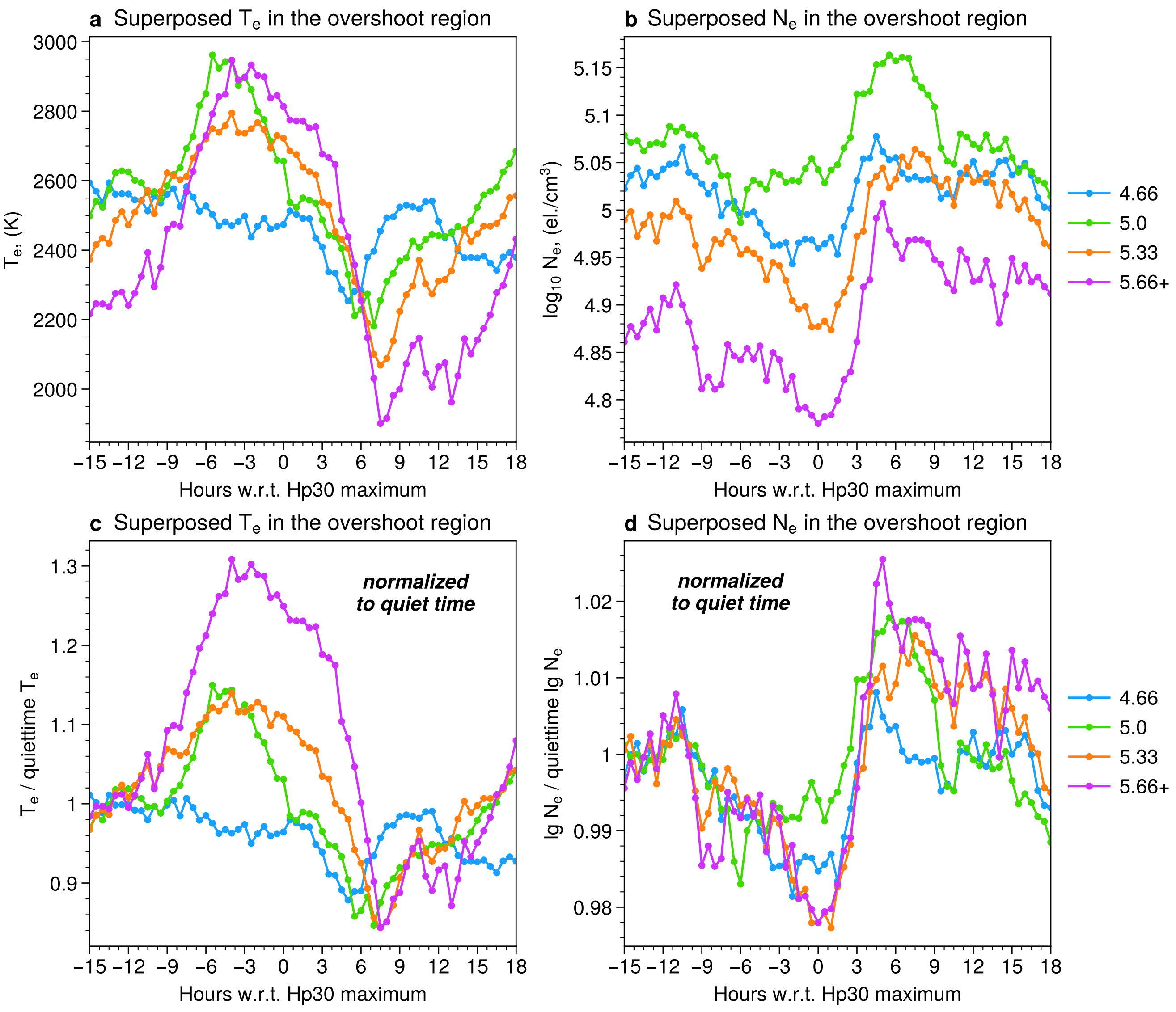

Figure S3. Storm-time variations of electron temperature and density (panels a and b, respectively). It can be seen that while the temperature values are roughly consistent in scale, electron density variations vary in terms of absolute scale (electron density values in the equatorial region can vary by several orders of magnitude). The bottom panels (c) and (d) show electron temperature and density values normalized to quiet time.

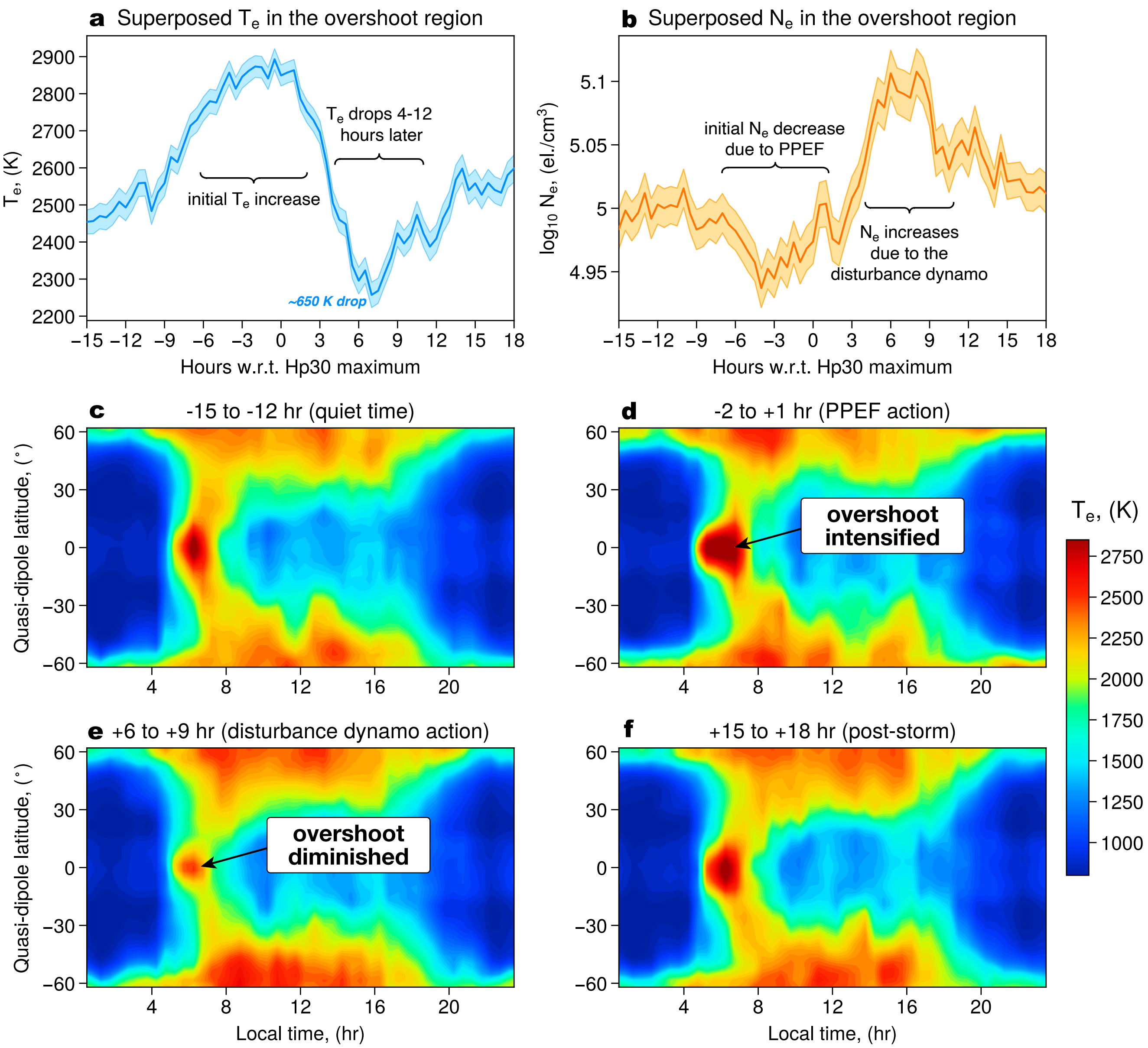

Figure S4. (a,b) Electron temperature and density values in the morning overshoot region ($|QDLat| < 5°$ and 05-07 LT) superposed for geomagnetic storms with Hp30>5. Lower panels show the superposed electron temperature distributions for the pre-storm (c), main phase (d), recovery (e) and post-storm (f) conditions. An intensification of the morning overshoot can be observed around the main phase, consistent with the depletion of N_e due to the action of PPEFs, and the suppression of the overshoot can be seen around 6-9 hours past the activity peak (panel e), consistent with the increase of N_e due to the action of disturbance dynamo electric fields.
